# Mid-term results and factors affecting outcome of a metal-backed unicompartmental knee design: a case series

**DOI:** 10.1186/1749-799X-4-39

**Published:** 2009-10-26

**Authors:** Thorsten M Seyler, Michael A Mont, Lawrence P Lai, Jipan Xie, David R Marker, Michael G Zywiel, Peter M Bonutti

**Affiliations:** 1 Department of Orthopedic Surgery, Wake Forest University School of Medicine, Winston-Salem, North Carolina, 27104, USA; 2 Rubin Institute for Advanced Orthopaedics, Sinai Hospital of Baltimore, Baltimore, Maryland, 21215, USA; 3 Department of Orthopaedic Surgery, Robert Wood Johnson School of Medicine, New Brunswick, New Jersey, 08903, USA; 4 Bonutti Clinic, Effingham, Illinois, 62401, USA

## Abstract

**Background:**

Controversies exist regarding the indications for unicompartmental knee arthroplasty. The objective of this study is to report the mid-term results and examine predictors of failure in a metal-backed unicompartmental knee arthroplasty design.

**Methods:**

At a mean follow-up of 60 months, 80 medial unicompartmental knee arthroplasties (68 patients) were evaluated. Implant survivorship was analyzed using Kaplan-Meier method. The Knee Society objective and functional scores and radiographic characteristics were compared before surgery and at final follow-up. A Cox proportional hazard model was used to examine the association of patient's age, gender, obesity (body mass index > 30 kg/m^2^), diagnosis, Knee Society scores and patella arthrosis with failure.

**Results:**

There were 9 failures during the follow up. The mean Knee Society objective and functional scores were respectively 49 and 48 points preoperatively and 95 and 92 points postoperatively. The survival rate was 92% at 5 years and 84% at 10 years. The mean age was younger in the failure group than the non-failure group (p < 0.01). However, none of the factors assessed was independently associated with failure based on the results from the Cox proportional hazard model.

**Conclusion:**

Gender, pre-operative diagnosis, preoperative objective and functional scores and patellar osteophytes were not independent predictors of failure of unicompartmental knee implants, although high body mass index trended toward significance. The findings suggest that the standard criteria for UKA may be expanded without compromising the outcomes, although caution may be warranted in patients with very high body mass index pending additional data to confirm our results.

**Level of Evidence**: IV

## Background

Unicondylar knee arthroplasty (UKA), in addition to total knee arthroplasty (TKA) and high tibial osteotomy (HTO), is a common surgical treatment for monocompartmental knee disease. The initial pain relief and function restoration achieved by UKA appear to be comparable to TKA and HTO [1]. Compared to TKA, the main perceived or real advantages of unicondylar knee arthroplasty include the preservation of bone stock, reduced incision size, and potentially more rapid recovery [2]. Furthermore, preservation of the posterior and anterior cruciate ligaments, the patellofemoral joint, and the meniscus in the unaffected compartment may help retain normal knee function [3]. In addition, there is typically less blood loss from the operation [4]. Compared to high tibial osteotomy, UKA appears to have a higher initial success rate and fewer complications [5].

However, the use of UKA has remained controversial since the 1970s because of differences in the success rates reported. Patient selection is believed to considerably influence the success of UKA [1,5]. As selection criteria continue to evolve, especially with improvements in surgical technique and UKA prosthetic design, the reliability of the outcomes with this procedure may improve.

The objective of this study was to examine the clinical and radiographic outcomes, the survivorship, and the predictors of failure of a metal-backed UKA design. The results from this study may lead to a better understanding of selection criteria for patients receiving UKA to help improve the outcomes of this procedure.

## Methods

### Study Design and Patient Demographics

**Figure 1:**
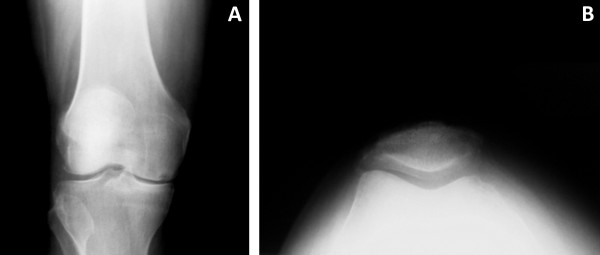
**Pre-operative antero-posterior (A) and Merchant view (B) radiographs of a patient with medial compartment osteoarthritis treated with a metal-backed unicompartmental knee arthroplasty**.

Sixty-eight patients (80 knees) treated with a metal-backed unicondylar knee prosthesis at our institution were followed prospectively. There were 39 women and 29 men, who had a mean age of 72 years (range, 44 to 91 years) and a mean body mass index (BMI) of 27 kg/m^2 ^(range, 17 to 39 kg/m^2^). The mean follow-up was 60 months (range, 24 to 168 months). Obese patients, defined as a BMI of 30 kg/m^2 ^or over, accounted for 28% of the cohort. The majority of the knees (n = 69, 86%) were diagnosed with osteoarthritis and the remainder (n = 11, 14%) were diagnosed with osteonecrosis. An overview of the patient demographics can be found in Table 1. The selection criteria for UKA included medial unicompartmental disease (Figure 1A) with intact cruciate ligaments, as evaluated during the pre-operative clinical consultation and confirmed intra-operatively. Patients with anterior knee pain, either as a clinical complaint or on pre-operative evaluation of knee extension against resistance, were deemed not appropriate candidates. Full institutional review board approval was granted for the investigation of these patients, all of whom provided written informed consent for participation in this study.

**Table 1:** Patient Characteristics

**Variables**	**Sample (n = 80)**
Mean age (years)	72 (44-91)
Men:Women (percent)	42:58
Mean body mass index (kg/m^2^)	27 (17-39)
Obesity (body mass index ≥ 30 kg/m^2^) (percent)	28
Pre-operative diagnosis (percent)	-
Osteoarthritis	86
Osteonecrosis	14
Follow-up period (months)	60 (24-168)
Unicondylar knee implant failure (percent)	11

### Clinical and Radiographic Evaluation

All patients were evaluated clinically and radiographically pre-operatively, as well post-operatively at approximately 3 months, 6 months, 1 year, and annually thereafter. Clinical evaluation was performed with use of the Knee Society (KSS) rating system [6], encompassing both objective and functional scores. Radiographic evaluation was performed using antero-posterior, lateral, and Merchant view radiographs of the knees (Figure 1B), with measurement of femoral and tibial angles, alpha and beta angles, and medial and lateral joint spaces as described by Villers and Cartier [7]. Patients were additionally evaluated for the presence of patellar osteophytes as an indicator of patellofemoral arthritis that is easily identifiable on most standard follow-up radiographs. Radiolucencies were evaluated at post-operative follow-up visits using the zone system described by Kennedy and White [8].

### Surgical Technique and Postoperative Management

All surgeries were performed by a single surgeon (P.M.B.) using a medial parapatellar approach. An M/G^® ^(Zimmer Inc., Warsaw Indiana) metal-backed unicompartmental prosthesis was used in all cases. Unicompartmental prostheses represent approximately 5% of the total number of knee arthroplasties performed by this surgeon in any given year. The skin incisions ranged from 10 to 15 centimeters. The patella was displaced laterally at the start of the procedure to inspect the patellofemoral joint and the lateral compartment, to evaluate the patella for the presence of osteophytes, and to confirm that the anterior and posterior cruciate ligaments (ACL and PCL) were intact. Inspection was done in both flexion and extension.

Varus releases were performed. Intramedullary instrumentation was used to make a distal femoral cut in 4 degrees of valgus orientation. The tibia was resected using an extramedullary alignment jig, with a minimum of 2 millimeters of bone removed in the greatest depth of deformity. A reciprocating saw was then used to make the center cut just medial to the ACL footprint and this bone fragment was removed. The leg was brought into extension to assess alignment. Next, femoral cuts were made and sized relative to the tidemark to avoid patellofemoral impingement. Finally, the chamfer, posterior, and peg cuts were made.

The tibia was sized in both the anterior-posterior (AP) and medial-lateral (ML) dimensions to optimize coverage while avoiding implant overhang. A keel cut and two peg cuts were made. Trial components were used to achieve 1 to 2 millimeters of laxity in full extension, with balanced flexion. Occasionally, additional soft tissue releases were required to achieve this aim.

**Figure 2:**
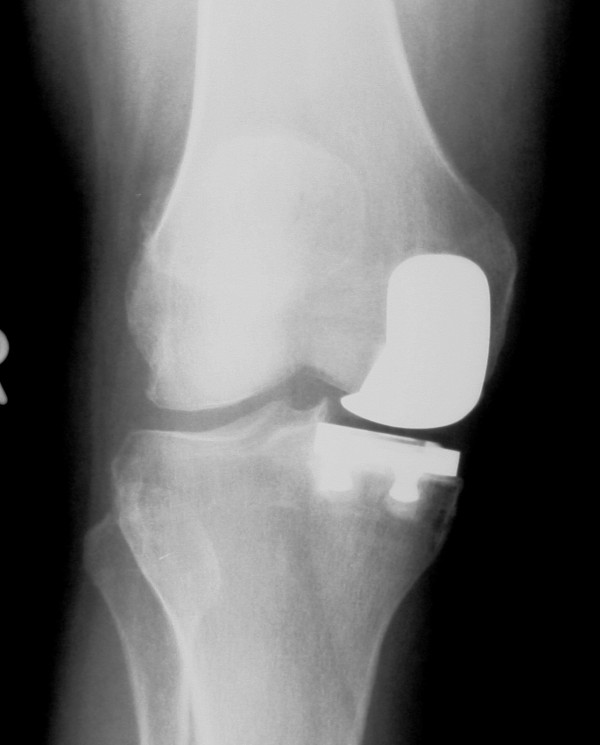
**Post-operative antero-posterior radiograph of the same patient shown in Figure 1 at 6 week follow-up visit**.

Next, the metal implants were cemented into position, starting with the tibial component followed by the femoral prosthesis. A polyethylene trial was then placed on the tibial tray, and the leg was brought into full extension to allow the cement to harden. The trial was then removed, and the residual cement was removed with an osteotome. The final polyethylene spacer was then implanted, and balancing and alignment of the knee was confirmed throughout the full range of motion of the knee (Figure 2).

### Data Analysis

Failure of UKA was defined as a revision to total knee arthroplasty (TKA). In our center we do not treat symptomatic aseptic loosening with implantation of a new UKA prosthesis; all these patients are revised to a TKA. The Kaplan-Meier method was used to estimate the survivorship of the prosthesis used in the study cohort. The Wilcoxon rank sum test was used to compare continuous variables (such as age, BMI, Knee Society scores, and most of radiographic measurements) between the failure and non-failure groups; a chi-squared or Fisher exact test was used to compare categorical variables (such as male, obesity (BMI ≥ 30 kg/m^2^), diagnosis, and presence of preoperational patella osteophytes) between the two groups. Similarly, Wilcoxon matched-pair signed-rank tests and chi-squared tests were used to compare continuous and categorical variables, respectively, before and after the operations. Seven factors were evaluated for association with implant failure: patient age, gender, obesity (BMI ≥ 30 kg/m^2^), pre-operative diagnosis (osteonecrosis or osteoarthritis), pre-operative Knee Society objective and functional scores, and the presence of patellar osteophytes prior to surgery. A Cox proportional hazard model was used to examine whether any of these factors were associated with the risk of failure of UKA, with a hazard ratio over one indicating that the factor was an independent predictor of a higher risk of failure of UKA. Patients that died or were lost to follow-up were excluded from this analysis.

## Results

### Clinical and radiographic outcomes

The mean preoperative Knee Society objective and functional scores were 49 points (standard deviation, SD = 9) and 48 points (SD = 10), respectively (Table 2). Both scores had substantially improved at final follow-up, with a mean of 95 (SD = 4) and 92 (SD = 7) points, respectively.

**Table 2:** Clinical and radiographic characteristics before and after UKA

**Variables**	**Pre-operative [SD]**	**Final follow-up [SD]**	**P value^a^**
Knee Society Scoring System			
Mean objective score (points)	49 [9]	95 [4]	< 0.01
Mean functional score (points)	48 [10]	92 [7]	< 0.01
Radiographic Characteristics			
Mean femoral angle (degrees)	97 [2]	97 [3]	< 0.01
Mean tibial angle (degrees)	84 [2]	84 [2]	0.81
Mean medial joint space (millimeters)	1.0 [1.0]	2.9 [1.6]	< 0.01
Mean lateral joint space (millimeters)	6.0 [2.0]	5.7 [1.9]	0.19
Mean patellar medial joint space (millimeters)	2.6 [1.6]	2.7 [2.0]	0.98
Mean patellar central joint space (millimeters)	3.6 [2.1]	3.2 [2.2]	0.04
Mean patellar lateral joint space (millimeters)	2.7 [1.9]	2.3 [1.8]	0.07
Presence of patellar osteophytes (percent of patients)	48	38	0.31
Alpha angle (degrees)	---	87 [4]	---
Beta angle (degrees)	---	82 [6]	---

SD = standard deviation

^a^p values were calculated based on Wilcoxon matched-pairs signed-ranks tests for continuous variables and chi-squared tests for categorical variables

Radiographic analysis revealed that the femoral angle increased by a mean of 0.9 degrees postoperatively (p < 0.01). However, there was no significant change in tibial angle. Medial joint space also increased significantly from a mean of 1.0 mm preoperatively to 3.0 mm postoperatively. At final follow-up, stable non-progressive lucent lines less than 2 mm in size were present in five of the unrevised patients (7%). One patient had a lucent line >2 mm in size but was asymptomatic and doing well at the most recent follow-up, with Knee Society pain and function scores of 92 and 90 points, respectively. One patient had progressive lucent lines in more than one zone and was judged to have an impending component failure. A revision was recommended to this patient but she refused as she was asymptomatic at most recent follow-up with Knee Society pain and function scores of 99 and 100 points respectively. A complete description of radiographic characteristics can be found in Table 2.

### Failure of UKA

**Figure 3:**
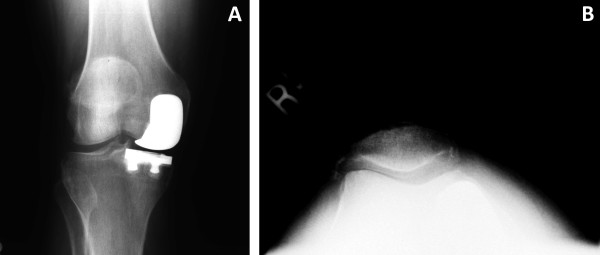
**Antero-posterior (A) and Merchant view (B) radiographs of the same patient as Figures 1 and 2, taken at 41 month follow-up**. The patient complained of increasing patello-femoral pain, and was revised to a total knee arthroplasty shortly thereafter.

**Figure 4:**
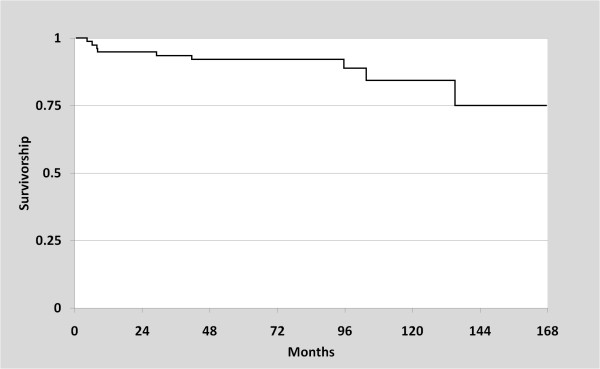
**Plot of Kaplan Meier survivorship estimate based on the failures of metal-backed unicompartmental knee arthroplasty components reported in the present study**.

Of the 80 knees that were treated with UKA, nine (11%) were revised to a TKA over the follow up period. Two cases were due to component loosening, and three were attributed to patellofemoral/lateral pain (Figures 3A and 3B). Other reasons for revision included polyethylene wear (n = 2), progression of arthritis (n = 1), and a tibial plateau fracture (n = 1). This tibial plateau fracture was non-traumatic in origin, and was likely due to implant subsidence into visibly osteopenic bone. The mean time from the date of UKA to revision to TKA was 48 months (range, 4 to 135 months). Kaplan-Meier survival analysis revealed that the survival rate of UKA implant was 92% at 5 years (95% CI: 83-96%), and 84% at 10 years (95% CI: 68-93%), found in Figure 4.

### Factors associated with failure of UKA

There were differences in some patient factors between the failure and non-failure groups, but no independent predictors of failure were identified. There was a significant difference in the mean age at index arthroplasty (73 versus 61 years; p < 0.01) between the non-failure and failure groups, respectively. There was a higher proportion of obese patients in the failure group compared to the non-failure group (44% versus 20%) but this difference was not significant (p = 0.11). Although the age difference was significant between the failure and non-failure groups, the hazard ratio of age was 0.94 (95% confidence interval, CI: 0.86-1.03), suggesting that age did not independently affect the risk of failure of UKA. Consistent with the descriptive analysis, obesity had a high hazard ratio of 2.12 but the 95% CI included a hazard ratio of 1.0. A more detailed comparison of the failure and non-failure groups can be found in Table 3.

**Table 3:** Comparison of patient characteristics between the failure and non-failure groups

	**Failure**	**Non-failure**		**Hazard Ratio**	
**Variables**	**(N = 9)**	**(N = 71)**	**P value^a^**	**Ratio**	**95% CI**
Mean age (years)	61 [8]	74 [9]	< 0.01	0.94	0.86-1.03
Male gender (percent)	44	42	1.00	0.30	0.05-1.87
Mean body mass index (kg/m^2^)	28 [7]	27 [4]	0.71	-	-
Obesity (body mass index ≥ 30 kg/m^2^) (percent)	44	20	0.11	2.13	0.34-13.3
Diagnosis (percent)				-	-
Osteoarthritis	100	85	0.35	-	-
Osteonecrosis	0	15		0.00	0.00
Mean pre-op objective score (points)	53 [10]	49 [9]	0.21	1.01	0.92-1.11
Mean pre-op functional score (points)	51 [5]	47 [10]	0.30	1.07	0.96-1.19
Pre-op patellar osteophytes (percent of patients)	33	46	0.38	0.27	0.07-2.01

CI = confidence interval

^a ^p values were calculated based on Wilcoxon rank sum tests for continuous variables, and chi-squared or Fisher exact tests for categorical variables.

## Discussion

Although patient selection is thought to influence the success of UKA, controversy remains over which specific factors affect the outcome of this procedure. Patient age, gender, and weight have been examined in previous studies without conclusive findings. Other factors, such as pre-operative diagnosis Knee Society function scores and patellar arthritis, have rarely been studied in relation to failure of UKA implant. This study used prospectively collected data to examine seven factors that may be associated with failure of UKA implants. We followed 80 knees for an average of 60 months. The survivorship of the UKA implants was 84% at 10 years follow up which is comparable to those reported in the literature [9,10]. Overall, we did not find any independent predictor of failure of UKA.

Traditionally, UKA was recommended for patients aged 60 years or over with a sedentary lifestyle [1]. However, with a hazard ratio of 0.94, our results suggest that age is not a predictor of failure of UKA. Gioe *et al*. examined the survival of 1,047 knee arthroplasties in patients aged 55 years old or younger using a community registry and did not find an association between age groups and survival rate [11]. Although the mean age in the failure group of the present study was 6 years younger than the non-failure group, young age was not found to be an independent predictor of failure. Several studies devote attention to younger patients (less than 60 years of age) treated with UKA, all of whom had excellent results. Schai *et al*. followed 28 knees in 28 patients who had a mean age of 52 years; only two knees were revised over a maximum of six years follow up [12]. Similarly, Pennington *et al*. reported a survival rate of 92% at 10 years in a group of younger patients [13]. Tabor and Tabor evaluated two patient cohorts to compare the survivorship and functional outcomes of UKA of patients aged 60 and over to those in a younger age group, and did not find a significant difference [10]. However, there are also studies reporting a poor survival rate in younger patients [14-16]. Additionally, using a Cox proportional hazard model, two studies found a hazard ratio of failure that favors superior outcomes in older patients [14,16]. The difference in these findings could be attributed to the age range of patients and the skills of the surgeons.

To date, gender has not been used as an inclusion/exclusion criterion for UKA, though some studies have found a difference in outcomes between male and female patients [10,17,18]. However, consistent with our findings, the majority of the studies did not find gender as a significant predictor of failure of UKA [11,14-16].

Weight and obesity are other factors to consider when UKA is applied. A multi-center investigation by Heck *et al*. reported mean BMIs in the failure and non-failure groups of 33 kg/m^2 ^and 25 kg/m^2^, respectively [17]. However, many other studies have not found an association between weight and/or obesity and failure of UKA [10,15]. One study even suggested that obese patients had a better survival rate when compared to their non-obese counterparts [18]. In addition, excellent survival rates have been reported in studies that did not consider weight when qualifying patients for UKA [19]. Despite some surgeons suggesting that patients over 80 kg or those who are clinically obese should not be treated with UKA [5,20], such criteria do not seem to be supported by the majority of studies, including the findings in the present report.

Although most UKAs are performed to in patients with osteoarthritis, it is not the only indication for UKA. Osteonecrosis can be treated with UKA with good results. Parratte *et al. *studied 31 osteonecrotic knees receiving UKA with a minimum follow up of three years and reported the survival rate of 96.7% at 12 years [21]. The authors noted that the outcomes of UKA were similar to those in primary osteoarthritis [12]. Similarly, Gioe *et al*. reported that there is no difference in survival rate based on diagnosis [11].

Preoperative Knee Society objective and functional scores, and patellar osteophytes have rarely been studied as predictors for UKA failure. Although anterior knee pain is a relative contradiction for UKA based on conventional surgical criteria, a recent study found that it did not affect the success of UKA using the Oxford phase 3 device [22]. Our findings indicate that pain and function of the affected knee are not related to failure of UKA. Patella osteophytes were also not a risk factor for UKA failure.

UKA is an effective treatment for unicompartmental knee disease. In addition to its clinical advantages, it may be more cost-effective when compared to TKA [23]. Opponents of UKA cite the poor survival rate of UKA implant relative to TKA. However, several studies have reported excellent survival rates [19,24]. Patient selection is a critical issue to success with this treatment modality. Conventional criteria suggest that patients should be over 60 years of age, weigh no more than 82 kg, and not perform heavy labor or be extremely physically active [20,25]. Although careful selection of patients is a key to the success of UKA, excessive restrictions will discount the benefits of the procedure and underplay its importance in treating unicompartmental knee disease. Better outcomes may be achieved with expanded criteria as the surgical technique and devices continue to be developed. Improvement in our understanding of factors related to UKA failure will shed light on patient selection criteria and help improve surgical outcomes of UKA.

Several limitations are noted in this study. First, the sample size is relatively small. Certain patient factors, notably obesity, trended towards significance in our analysis of independent predictors of failure, and it is possible that a larger study group would provide additional power to better define the associations between the factors and risk of failure of UKA. Additionally, because of the small and diverse number of failures, we did not attempt to assess hazard ratios for each individual cause for revision. It is possible that such an analysis would reveal variability in independent associations for some modes of failure. Finally, the follow up time is relatively short compared to some other studies on UKA. The average length of follow up was five years, which affects the survival rate in this study. In addition, long-term outcomes could not be assessed.

## Conclusion

Young age, gender, obesity, diagnosis, pre-operative objective and functional scores and patella osteophytes were not predictors of failure of a unicondylar knee implant, although increased obsesity was association with a high hazard ratio. The findings suggest that the standard criteria for UKA may be expanded without compromising the outcomes, although caution may be warranted in patients with very high body mass index pending additional data to confirm our results.

## Competing interests

No external financial support was received in support of this study.

MAM is a consultant for Stryker Orthopaedics and Wright Medical Technologies, and receives royalties from Stryker Orthopaedics. PMB is a consultant for Stryker Orthopaedics, and receives royalties from Stryker, Arthrocare, Biomet, and Synthes.

None of the other authors have any financial or non-financial competing interests to disclose.

## Authors' contributions

TMS, MAM, LPL, JX, PMB designed the study. LPL, DRM, PMB collected the data. TMS, LPL, JX, DRM, MGZ, analyzed the data. TMS, MAM, DRM, MGZ, prepared the manuscript. MAM, JX, MGZ, PMB ensured the accuracy of the data and analysis. All authors have read and approved the final manuscript.
